# A Study on High-risk Premarital Sexual Behavior of College Going Male Students in Jamnagar City of Gujarat, India

**DOI:** 10.5812/ijhrba.11855

**Published:** 2013-12-12

**Authors:** Viral R Dave, Naresh R Makwana, Babusingh S Yadav, Sudha Yadav

**Affiliations:** 1 Department of Community Medicine, GCS Medical College, Hospital and Research Centre, Ahmedabad, India; 2 Department of Community Medicine, M.P. Shah Medical College, Jamnagar, India; 3 Department of Community Medicine, Gujarat Adani Institute of Medical Sciences, Bhuj, India

**Keywords:** Sexual Behavior, Disorders of Sex Development, Condoms

## Abstract

**Background:**

The pre-marital sex and live-in relationship among young people are increasing at an alarming rate. Remote consequences of such high risk behaviors are increase in the incidence of STDs (including HIV), unsafe and illegal abortion, adolescent pregnancy and motherhood, single mother child/abandoned child, juvenile delinquency and many more.

**Objectives:**

The aim of this study was to investigate the high-risk sexual behaviors in depth, influenced by various factors including age at sexual debut, type of partners, consistent condom usage, hostel stay, socioeconomic class, etc. among college-going male youth.

**Materials and Methods:**

The study was conducted in Jamnagar among undergraduate (18-24 years) male college students. A total of 450 students were randomly selected from three colleges of Jamnagar.

**Results:**

Out of all 450 participants, 49.11% were in the age group of 18-20 years. Among study subjects, 13.78% had one or more pre-marital sexual exposures. In students with positive pre-marital sexual history, the various sex partners were girlfriends (95.16%), commercial sex workers (14.5%), homosexuals (6.45%), and multiple sex partners (33.88%). Among students, 62.9% were using condom consistently. Three-fifth of the ones indulged in premarital sex, were in the age group of 16-20 at the time of sexual debut.

**Conclusions:**

Most of the students were quite young (16-18 years) at the time of first pre-marital sexual exposure. Consistent condom usage was not uniform. The students staying at hostels, indulged in premarital sex, were found to have multiple sex partners.

## 1. Background

The present young generation of India is following life-style of their counterparts in the western culture. There is a competitive behavior among young college-going youth to prove themselves as more modern, impressive and so-called high-cultured people.

Young people mature earlier but marry later. So many of them start sex at a younger age and may have multiple partners including sex workers. The repercussion of pre-marital sex in terms of health-related or psychosocial problems is hardly affecting the young people who are the potential growth engine of the country.

Pre-marital sex and live-in relationship among young people are increasing at an alarming rate. The young generation is becoming a prey of this social evil and facing the remote consequences of such high risk behaviors which are increase in the incidence of sexually transmitted diseases (STDs) (including HIV), unsafe and illegal abortion, adolescent pregnancy and motherhood, single mother child/abandoned child, juvenile delinquency and many more.

At present, the Government and various non-governmental organizations (NGOs) working in the field of health are concentrating towards particular communicable and non-communicable (life-style) diseases; but the ongoing psycho-social problems in the country due to the increasing trend of experimentation–“taking a chance” behavior are undermined, especially among male youth. In such relationships, males are considered to possess the decision-making and dominating roles, as far as this situation is concerned. The reported rate of students engaging in pre-marital sex in different region of India varies from 8% to 15% (1).

This behavior is creating a new cultural facet in India by people living with the above mentioned problems in society. The weapons to fight such enemies of social health are yet to be discovered.

## 2. Objectives

The aim of this study was to investigate the high-risk sexual behaviors in depth, influenced by various factors including age at sexual debut, type of partners, consistent condom usage, hostel stay, socioeconomic class, etc. among college-going male youth.

## 3. Materials and Methods

The study was conducted in Jamnagar city of India among undergraduate (18-24 years) college going male students from January to June 2009. Sample size was calculated according to the given guideline in surveillance of HIV risk behaviors, participant manual module 5 (2). It was calculated to be 429, but to round off the analysis it was considered 450.

Jamnagar has nine colleges providing various undergraduate courses. Among them, three colleges were randomly chosen. One hundred and fifty students were selected by systemic randomization from each of the three colleges. It was emphasized to select almost equal number of representatives from each year of their study courses. The research permission was issued by the college authorities. Informed consent was taken from the participants.

Initially, a pilot study was performed in one college with 50 male students. After analyzing the result, a modified proforma was prepared. The young students were introduced about objectives of the study and reassured that personal information would be kept confidential. In each session, 20 students were placed in one classroom and any kind of discussion within the students was not allowed.

The questionnaire comprised general information about the students their sexual behavior in detail, including their age at first pre-marital sex, multiple sexual partners, STDs and consistent condom usage.

## 4. Results

Out of 450 total participants, 221 (49.11%) were in the age-group of 18-20, 38% in 20-22, and 12.89% ≥ 22 years; 82.22% of participants came from an urban background and 30.22% belonged to higher socioeconomic classes, while 49.11% and 20.67% were from middle and lower socioeconomic classes, respectively. At the time of survey, 437 (97.11%) students were un-married and 101 (22.44%) had hostel stay history of any duration within the past 5 years (Table 1), 69.30% of which was in a 1-3 years duration and 11.89% more than 5 years. 

**Table 1.** Socio-demographic Profile of the Participants (n = 450)

Variables	No. (%)
**Age, y**	
18-20	221 (49.11)
20-22	171 (38)
≥ 22	58 (12.89)
**Residence**	
Urban	370 (82.22)
Rural	80 (17.78)
**Socio-economic status**	
Higher	136 (30.22)
Middle	221 (49.11)
Lower	93 (20.67)
**Marital status**	
Married	13 (2.89)
Unmarried	437 (97.11)
**History of hostel stay**	
Yes	101 (22.44)
No	349 (77.56)

**Figure 1.**
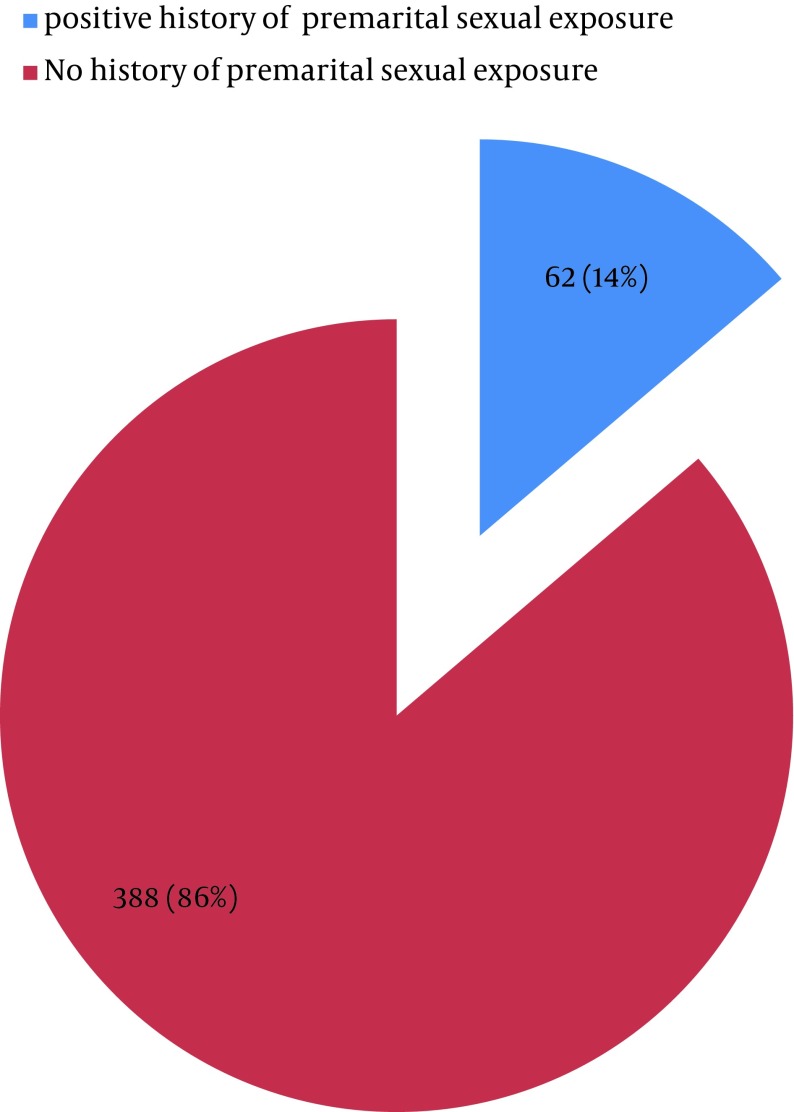
Distribution of Students According to History of any Sexual Exposure Before Marriage

Out of 450 total studied cases, 62 (13.78%) had one or more pre-marital sexual exposures (Figure 1). Among them, 59 (95.16%) had pre-marital sex with their girlfriends while 14.5% had one or more sexual relations with commercial sex workers (CSWs); 4 (6.45%) participants were involved in homosexual activities and 21 (33.88%) had multiple sex partners. 

**Figure 2.**
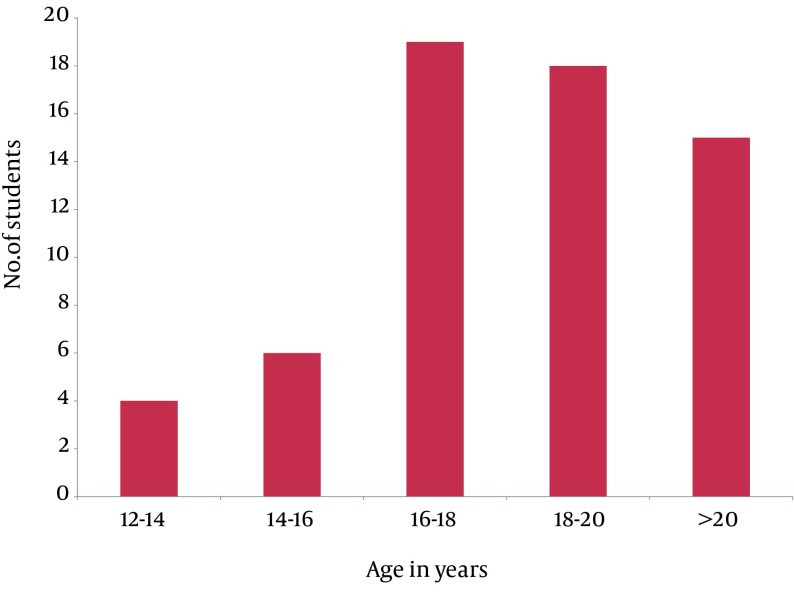
Distribution of Students According to “Age at premarital sexual debut”

Majority (about 60%) of students indulged in premarital sexual activities, were in the age group of 16-20 at the time of their sexual life initiation, while 24.18% were > 20, 16.13% were below 16, and unfortunately, four students were only 12-14 years (Figure 2). 

**Figure 3.**
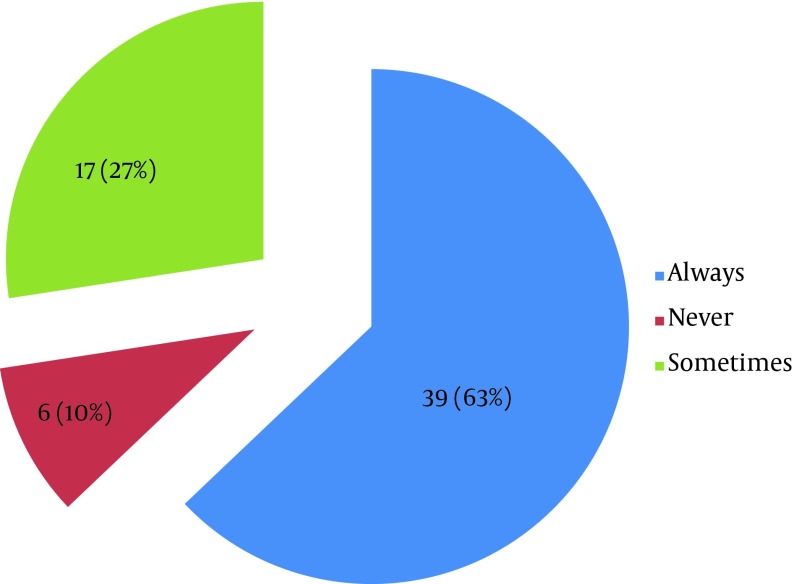
Distribution of Students According to Whether They Used Condom Regularly or Not

Out of 62 total participants with positive premarital sexual exposure, 39 (62.90%) used condom during their first premarital coitus while 23 (37.10%) did not, meaning that they were taking the risk of STDs including HIV in their very first sexual exposure. Among students who indulged in premarital sexual relations, 39 (62.9%) regularly, 27.4% sometimes, and 9.68% never used condom (Figure 3). 

Out of 62 total participants, 4 (6.45%) had history of STD after sexual exposure, 50% of which did not take any kind of treatment. Considering association between ages of premarital sexual debut and use of condom, it was found that the condom usage increased with the growth of students’ ages. The result was statistically significant (P = 0.0257). Out of five total students in the age group of < 15, none of them used condom at their first sexual experience, but in the age group of 15-17, almost 2/3 of them used it (Table 2). 

**Table 2.** Association Between the Age of Student at Their First Sexual Act and Use of Condom^a^

Age, y	Use of Condom, No. (%)		Total, No. (%)
	Yes	No	
**< 15**	0 (0.0)	5 (100)	5 (100)
**15-17**	16 (66.67)	8 (33.33)	24 (100)
**18-20**	18 (69.23)	8 (30.77)	26 (100)
**> 20**	5 (71.42)	2 (28.58)	7 (100)
**Total**	39	23	62

^a^ Chi-square: 9.288; P = 0.0257.

Surveys on the association between two high-risk behavioral characteristics including multiple sex partners and regular use of condom, revealed that only 42.85% of participants with multiple sex partners were using condom at each exposure; meaning that young people taking the risk of multiple sex partners, were also ignorant of consistent condom usage. Among students with single sex partners, 73.17% used condom consistently; meaning that they are quite protective by having single partners as well as regularly using condom (Table 3). 

**Table 3.** Distribution of Multiple Sex Partners Students Association With Their Condom Usage at Each Exposure^a^

Students With Multiple Sex Partners	Using Condom at Each Exposure, No. (%)		Total, No. (%)
	Yes	No	
**Yes**	9 (42.85)	12 (57.15)	21 (100)
**No**	30 (73.17)	11 (26.83)	41 (100)
**Total**	39	23	62

^a^ Chi-square: 4.247; P = 0.0393.

Among the upper-class students with history of premarital sex, 78.26% used condom at their first sexual exposure, while it was 64% for the middle class and only 35.71% for the low socio-economic class. It was observed that use of condom at first sexual exposure was on a decreasing trend from the upper to the lower class. The result was statistically significant (Table 4). 

**Table 4.** Association Between Socio-economic Class of the Participants and Use of Condom at Their First Sexual Exposure ^a^

Socio-economic Class	Use of Condom at the First Sexual Exposure, No. (%)		Total, No. (%)
	Yes	No	
**Upper**	18 (78.26)	5 (21.74)	23 (100)
**Middle**	16 (64.00)	9 (36.00)	25 (100)
**Lower**	5 (35.71)	9 (64.29)	14 (100)
**Total**	39	23	62

^a^ Chi-square: 6.773; P = 0.0338.

Analyzing the association between residence region and involvement in premarital sex, it was observed that 11.89% were from urban backgrounds while 22.5% (almost double) belonged to rural residencies. The association was statistically significant with P value = 0.0205.

The association between hostel stay and tendency for multiple partners was also analyzed. Among the students staying at hostels who indulged in premarital sex, 57.90% had multiple partners while among others, it was 23.26%. The result was also statistically significant with P value = 0.0180.

## 5. Discussion

The national pre-marital sex ratio (3) among male youth is around 15-16%. Rachna Sujoy (4) in her similar study among youth of Gujarat, found that 16.4% of male youth had premarital sexual intercourses. Abraham et al. (5) in their study among unmarried youth of Mumbai colleges reported the premarital sex rate as 26%. It may be higher compared to the present study due to high westernization of the social culture in Mumbai. Jejeebhoy (6) reported 9% and 25% prevalence of premarital sexual activities among adolescent boys of Gujarat and Delhi, respectively.

In the similar study by the national AIDS research institute (NARI) among undergraduates from six colleges of Pune (7), 37% of boys were reported to have premarital heterosexual experiences, 4% sex with CSWs, and 15% homosexual experiences. Only one third of students with history of sexual exposure were reported to have condom usage and even a fifth of them its consistent use. Sathe AG and Sathe S (8) found that prevalence of premarital sex was 22% among boys. In the same study, findings about sex partners’ distributions were sex workers (13.9%), relatives (8.3%), neighboring girls (36.1%), girlfriends (43.8%) and married women (14.8%). Jaya and Michelle J Hindin (9) in their study at Delhi, found that 32% of males had sex with the opposite sex while 10% had homosexual experiences. Tivvari et al. (10) in their study among youth of Delhi, found that 5.1% of males had homosexual relations (men who have sex with men or MSM), 17.9% had sex with the opposite gender, 8.6% visited CSWs, and 3.3% had multiple sex partners; while in a same study at Lukhnow, the results were: 5.1% were MSM, 25.7% had relation with the opposite gender, 11.3% visited CSWs and 3.6% had multiple partners. In the present study, 95.16% of cases had premarital sex with their girlfriends, while 14.5% had one or more sexual relations with CSWs.

According to the national family health survey-3 (NFHS-3) (11), at the time of the survey, among the age group of 15-19 years, 2.7% experienced their first sexual intercourse before the age of 15 and among 20-24 age group, 1.8% had it before 15 and 11.2% before 18. In this survey, out of the total studied cases (15-24 years) with positive premarital sexual history, 18.5% of 15-19 and 14.1% of 20-24 age groups used condom at their first sexual intercourse. Kamtchoning et al. (12) studying Cameroonian students found that 52% were sexually active, among which 56% experienced their first sexual encounter between the ages of 15 and 17. Mclean (13) researching the high school students of Swaziland found that they were sexually active by the age of 16. In the present study, among students with positive premarital sex history, 60% started their sexual relations between 16 to 20 years.

In a behavioral surveillance survey performed in Maharashtra in 2001, among 15-24 years unmarried youth of Slums, the findings were: 71% of respondents always used condoms for CSWs, but only 33% consistentlyused it for noncommercial partners. Among youth aging 20-24 years, 66% reported consistent condom usage during commercial sexes, but only 30% for noncommercial partners.

Adhikari and Tamang (14) in their study at Nepal, found that about 57% of male students used condom at their first sexual intercourse. Sachdev P (15) in a similar study found that only four in ten students from Delhi University reported occasional condom usage during sexual intercourses. Rachna Sujoy (4) in her similar study found that 30.3% of sexually active unmarried young males consistently used condom. In the present study, 62.9% of students regularly used condom at each sexual exposure while 9.68% had never used condom.

Jeejibhoy and Sebastien (16) found growing evidences of premarital onset of sexual activity, particularly among young males from urban areas. A study in Maharashtra (17) revealed that rural youth were twice as likely as their urban counterparts to have experienced premarital sex (21% of young men in rural areas compared to 11% in urban areas). Similar finding was seen in the present study.

In the information era, young generations are getting early puberty, but without psychological maturity. The age of sexual debut is also decreasing as low as 12-14 years. Socio-economic class plays its own role; upper class students have more prevalence of premarital sex and consistent condom usage, while lower socio-economic class students have more prevalence of multiple sex partner and occasional condom usage. The prevalence of indulgence in premarital sex was more in students staying at hostels compared to the ones not staying at hostels. Even among hostel-staying students, the incidence increased with longer hostel accommodation, which might be due to the fact that they still hesitate to follow such high risk behaviors when they are in custody of parents, despite living in mixed cultures. Under the effects of western acculturation in our country, the rate of premarital sex indulgence by young people is on an increasing trend. So the actions such as easy and continuous condom availability development, especially around colleges, hostels and campuses, repeated generation awareness programs at colleges and hostels, availability of youth counselors at colleges, and promoting diagnostic and treatment facilities for sexually transmitted infections (STIs) should be strongly considered.

## References

[1] (1996). A study on youth frustration with special emphasis on college students..

[2] WHO (2007). Surveillance of HIV Risk Behaviours,Participant Manual Module 5..

[3] Youth in India, Situation and Needs..

[4] Sujoy R (2006). RPremarital Sexual Behaviour among Unmarried College Students of Gujarat, India; Health and Population Innovation Fellowship Programme..

[5] Abraham L, Kumar K A (1999). Sexual experiences and their correlates among college students in Mumbai City, India.. Int Famil Plan Perspect..

[6] Jejeebhoy SJ (1998). Adolescent sexual and reproductive behavior: a review of the evidence from India.. Soc Sci Med..

[7] NARI (National AIDS Research Institute) Pune (1997). The Youth sexuality study at Pune University.. http://www.nari-icmr.res.in/completed_projects.html.

[8] Sathe AG, Sathe S Knowledge, Behaviour and attitudes about sexuality amongst adolescents in Pune: A situational analysis.. edind.nic.in/jah/t05/i1/jaht05i1p49.pdf.

[9] Jaya J, Hindin MJ (2009). Premarital romantic partnerships: attitudes and sexual experiences of youth in Delhi, India.. Int Perspect Sex Reprod Health..

[10] Tivvari VK, Kumar A (2004). Premarital Sexuality and Unmet Need of Contraception among Youth-Evidence from two cities of India.. J Family Welfare.

[11] (2005.). National Family Health Survey-3. Ministy of Health & Family Welfare,Govt. of India..

[12] Kamtchouing P, Takougang I, Ngoh N, Yakam I (1997). Sexuality of adolescent students in Yaounde (Cameroon).. J Contracept Fertil Sexual..

[13] McLean PE (1995). Sexual behaviors and attitudes of high school students in the kingdom of Swaziland.. J Adolesc Res..

[14] Adhikari R, Tamang J (2009). Premarital sexual behavior among male college students of Kathmandu, Nepal.. BMC Public Health..

[15] Sachdev P (1998). AIDS/HIV and university students in Delhi, India: knowledge, beliefs, attitudes and behaviors.. Soc Work Health Care..

[16] Jejeebhoy J, Sebastian MP (2013). Actions that protect: Promoting sexual and reproductive health and choice among young people in India..

[17] International Institute for Population Sciences (IIPS) and Population Council. (2). A study on Romance and sex before marriage among young women and men in Maharashtra, Youth in India Situation and Needs 2006–2007.. http://www.popcouncil.org/pdfs/2008PGY_YouthInIndiaBriefRomanceMa.pdf.

